# Traumatic Brain Injury in the Omics Era: Plasma and Extracellular Vesicle Proteomic Signatures in Polytrauma

**DOI:** 10.3390/medsci14040429

**Published:** 2026-07-25

**Authors:** Liudmila Leppik, Birte Weber, Cora R. Schindler, Louise Funda, Marcus Krüger, Sebastian Proschinger, Dirk Henrich, Ingo Marzi

**Affiliations:** 1 Department of Trauma Surgery and Orthopedics, University Hospital, Goethe University Frankfurt, 60590 Frankfurt, Germany; bi.weber@med.uni-frankfurt.de (B.W.); schindler@med.uni-frankfurt.de (C.R.S.); louise.funda@googlemail.com (L.F.); sebastian.proschinger@tu-dortmund.de (S.P.); d.henrich@trauma.uni-frankfurt.de (D.H.); marzi@trauma.uni-frankfurt.de (I.M.); 2 Institute for Genetics, Cologne Excellence Cluster on Cellular Stress Responses in Aging-Associated Diseases (CECAD), 50931 Cologne, Germany; marcus.krueger@uni-koeln.de; 3 Research Group “Sports Medicine”, Institute for Sport and Sport Science, TU Dortmund University, 44227 Dortmund, Germany

**Keywords:** traumatic brain injury, extracellular vesicles, biomarkers, proteomic, polytrauma

## Abstract

**Background/Objectives**: Clinical outcomes after traumatic brain injury (TBI) remain difficult to predict, highlighting the need for more sensitive diagnostic and prognostic biomarkers, particularly in polytrauma. This study aimed to identify TBI-specific proteomic signatures in plasma and extracellular vesicles (EV)-enriched fractions of critically injured trauma patients. **Methods**: Seventy-five severely injured adult trauma patients (ISS ≥ 16) were included: isolated severe TBI (TBI; AIS_head_ ≥ 4, other AIS ≤ 1, *n* = 23), polytrauma with TBI (PT-TBI; AIS_head_ ≥ 4, *n* = 22), and polytrauma without TBI (PT; AIS_head_ = 0, *n* = 30). 24 age- and sex-matched healthy volunteers served as controls. Neat plasma and EV-enriched fractions were profiled using HPLC-MS/MS. Differentially expressed proteins (DEP) were analyzed bioinformatically, and associations with clinical parameters were assessed using Spearman’s correlation. **Results**: The EV-enriched plasma fraction yielded more DEPs than neat plasma (846 vs. 258). Most DEPs were linked to polytrauma, with plasma reflecting metabolism and EVs showing translation and protein catabolism signatures. Among TBI-context proteins, EV-associated NRCAM and AQR and plasma IGHV1-69D, MASP1, PON1, and IGFBP7 remained significantly associated with TBI after adjustment for confounder. Notably, only EV-associated proteins correlated with injury-related clinical parameters. AQR negatively correlated with GCS (*r* = −0.51, *p* < 0.0001), while elevated NRCAM, AQR, and plasma MASP1 were associated with neurological deterioration and neurosurgical intervention. **Conclusions**: EV-enriched plasma proteomics enhances biomarker discovery in polytrauma, revealing distinct pathways and greater sensitivity than whole plasma. While most changes reflect systemic injury, EV-associated NRCAM and AQR, along with plasma MASP1, show TBI-specific associations with neurological status, highlighting their potential as candidate TBI biomarkers for further study.

## 1. Introduction

Polytrauma (PT) remains to be a major global health challenge, accounting for approximately 4.4 million deaths annually and causing significant morbidity across all age groups [1]. Young males experience trauma twice as often as other demographic groups; however the incidence of trauma among individuals over 65 is increasing, as is the prevalence of severe traumatic brain injury (TBI) [2]. Significant advances have been made in last decades in both prevention (e.g., vehicle and workplace safety) and clinical management of PT [3,4,5,6] and in-hospital mortality has decreased from 40–50% in the 1970s to 12% as of 2023 (Annual Report TR-DGU^®^). Today, PT patient outcomes are primarily determined by TBI (46% mortality), followed by hemorrhagic shock and organ failure [7]. Despite decades of research into the complex interactions between TBI and polytrauma, the effect of traumatic brain injury on multiple trauma outcomes is still not well understood. Poor outcomes in both polytrauma and TBI are driven by diverse pathological mechanisms, including excessive inflammation, impaired coagulation, endothelial dysfunction, and other systemic disturbances [8,9,10].

Diagnosis and treatment of TBI in the context of polytrauma are particularly challenging, as TBI pathology can be masked by polytrauma-and vice versa-thereby driving the search for robust biomarker capable of reliably detecting TBI in the setting of polytrauma. With recent advances in technological development, biomarker discovery strategies have shifted from the analysis of individual molecules [11,12,13] to comprehensive, system-level omics approaches [14]. Thus, recent large-scale proteomic, metabolomic, and lipidomic studies on polytrauma patients have shown that individual differences in trauma response, even among patients with similar injury patterns, can influence clinical outcomes [15]. Comparison of polytrauma patients with and without TBI revealed an enrichment of coagulation cascades and neuronal markers among plasma proteins, as well as association of TBI with elevated glycolytic metabolites and conjugated bile acids [16]. However, no proteins whose expression was significantly and specifically dysregulated in any of the patient groups (polytrauma with TBI, polytrauma without TBI, or isolated TBI) was identified, which was attributed to limitations in the analytical techniques used.

Growing exploration of the field of extracellular vesicles (EV) and increasing recognition of their importance as lipid membrane–protected reservoirs of bioactive molecules have stimulated the search for potential biomarkers and injury-specific signatures within this compartment. It has been shown that EVs bearing epitopes derived from their parental cells can reflect specific injuries, such as TBI, spinal cord injury (SCI) and others [17,18,19]. Moreover, the molecular cargo of EVs appears also to reflect the trauma type and have prognostic potential [20,21].

Based on this, the primary objective of this pilot study was to characterize and compare the proteomic profiles of the EV-enriched plasma fraction and total plasma in patients with isolated TBI, polytrauma with TBI, polytrauma without TBI, and healthy controls. A secondary objective was to identify candidate proteins associated with TBI in the context of polytrauma and to explore their associations with clinical parameters, including neurological status and injury severity. We hypothesized that the EV-enriched plasma fraction contains distinct molecular signatures compared with total plasma and may provide complementary information for the identification of TBI-associated proteomic changes in complex trauma settings.

## 2. Materials and Methods

### 2.1. Ethics, Patients and Sample Collection

The retrospective study was conducted at the Level 1 Trauma Centre (the University Hospital of Goethe University, Frankfurt am Main, Germany) following approval by the local Institutional Review Board (approval number 94/19, issued on 4 September 2019). Written informed consent was obtained from all participants or from their legal representatives.

75 severely injured adult trauma patients (age ≥ 18; ISS ≥ 16) were included in the study and subdivided in three groups: (1) isolated severe TBI (TBI; AIShead ≥ 4 and all other AIS ≤ 1, *n* = 23); (2) polytrauma with TBI (PT-TBI; AIShead ≥ 4, *n* = 22), and (3) polytrauma without TBI (PT; AIShead = 0, *n* = 30). 24 age- and sex-matched healthy volunteers served as control group. Patients included in this study were selected from the trauma registry according to predefined clinical inclusion criteria and the availability of suitable biological material for proteomic analysis.

Blood was collected at the admission to the emergency room (ER, <3 h after trauma), plasma was gained via centrifugation (3000× *g*, 15 min, 4 °C), aliquoted and stored at −80 °C until analysis.

### 2.2. Proteome Profiling

#### 2.2.1. Sample Preparation

Prior to proteomic analysis, all samples were anonymized and assigned unique identification codes, and sample processing and measurement were performed using these coded samples. For neat plasma analysis, plasma samples were prepared following a modified SP3 protocol [22] using the Microlab STAR Liquid Handler (Hamilton Company, Reno, NV, USA) as a fully automated Robotic Liquid Handling System. In brief, cryopreserved plasma was thawed at 4–8 °C and transferred to a PCR plate. Plasma was diluted (1:20) to 100 µL with a lysis buffer (50 mM TEAB, 1% SDS, 1% SDC) and then reduced and alkylated (12.5 mM TCEP, 50 mM CAA) for 20 min at 60 °C and 600 rpm. To induce on-bead protein precipitation, the volume was adjusted to 70% acetonitrile (ACN) and 20 µL of equilibrated hydrophilic and hydrophobic Sera-Mag Magnetic Carboxylate beads at a ratio of 1:1 were added, followed by shaking for 15 min at 400 rpm at RT. On a magnet, beads were washed twice with EtOH and once with ACN, dried for two min and digested on beads in 40 µL 50 mM TEAB buffer comprising trypsin (3.5 µg; SERVA Electrophoresis GmbH, Heidelberg, Germany) for 5 h at 37 °C and 400 rpm. After digestion, 20 µL of buffer R (2% acetonitrile and 5% formic acid in water) was added and on-magnet, the protein digest was gathered, transferred to a new plate, quenched to 0.1% formic acid and frozen at −20 °C until analysis. Immediately before analysis, 250 ng of peptides were loaded on EvoTips according to the manufacturer recommendations (Evosep Biosystems, Odense, Denmark).

In the second approach, the method described by Wu et al. was used to simultaneously enrich plasma-derived extracellular vesicles and deplete abundant plasma proteins [23]. For sample preparation, the ASSIST PLUS pipetting robot (Integra Biosciences, Zizers, Switzerland) was used as a semi-automated Robotic Liquid Handling System. The protocol described by Wu et al. (2025) [23] was followed with minor adaptations, including semi-automated sample handling, alkylation with chloroacetamide, overnight on-bead digestion using trypsin/LysC, and omission of the ethanol washing steps. In brief, cryopreserved plasma was thawed at 4–8 °C, 50 µL were transferred to a Nunc™ 1 mL DeepWell™ plate and mixed with 50 µL binding buffer (100 mM Bis-Tris Propane, pH 6.3, 150 mM NaCl). Then, 12.5 µL equilibrated MagReSyn^®^ strong anion exchange beads (ReSyn Biosciences, Edenvale, South Africa) were added and incubated on a ThermoMixer^®^ C (Eppendorf SE, Hamburg, Germany) for 45 min at RT and 800 rpm. Beads were washed three times with 500 µL wash buffer (50 mM Bis-Tris Propane, pH 6.3, 150 mM NaCl) for 5 min at RT and 800 rpm, then lysed and reduced (50 mM Tris, pH 8.5, 1% SDS, 10 mM TCEP) for 60 min at 37 °C and 1000 rpm. Proteins were alkylated by adding 15 mM chloroacetamide for 30 min at RT and 800 rpm. To induce on-bead protein precipitation, the volume was adjusted to 70% ACN, mixed and incubated for 10 min without shaking. Beads were washed three times with 500 µL 95% ACN on magnet. On-bead digestion (overnight, 16 h) at 37 °C and 800 rpm was done in 200 µL 50 mM ammonium bicarbonate buffer comprising trypsin (750 ng, SERVA Electrophoresis GmbH) and LysC (200 ng, KPL ApS). The digestion was then quenched to 0.5% trifluoracetic acid. After placing the plate on a magnet for 3 min, the protein digest was gathered, and samples were frozen at −20 °C until analysis. Immediately before analysis, 250 ng of peptides were loaded on EvoTips according to the manufacturer recommendations (Evosep Biosystems).

#### 2.2.2. HPLC-MS/MS Analysis

Samples were injected using the 60 SPD (neat plasma samples) or 30 SPD (EV-enriched samples) chromatography method on an EvoSep One system coupled to an 8 cm × 150 µm (neat plasma samples) or 15 cm × 150 µm (EV-enriched samples) (1.5 µm particle size) PepSep C18 column (both Evosep Biosystems) with the column temperature maintained at 50 °C using an integrated column oven. The sequence of sample acquisition was conducted in a randomized order irrespective of group allocation. Peptides were separated using a binary buffer system compromised of 0.1% FA in MS-grade water as solvent A and 0.1% FA in MS-grade ACN as solvent B on a 20 cm × 550 mm analytical column (nanoViper™, Thermo Fisher, Waltham, MA, USA). The HPLC system was coupled to a timsTOF pro 2 using a CaptiveSpray source (20 µm) (both Bruker Daltonics, Bremen, Germany). Samples were measured in dia-PASEF mode with daily ion mobility calibration using three ions in the Agilent ESI-Low Tuning Mix following the vendor’s specifications. The DIA-PASEF windows ranged in dimension from 350 to 1250 *m*/*z* and 1/k0 0.7–1.45, with 2 × 12 Th windows (neat plasma samples) or 2 × 16 Th windows (EV-enriched samples) (py-DIA based window-sizing) [24] for proteomics analysis. All scans were acquired in positive ionization mode, and the collision energy was ramped linearly as a function of the mobility from 59 eV at 1/K0 = 1.6 V.s/cm^2^ to 20 eV at 1/K0 = 0.6 V.s/cm^2^.

#### 2.2.3. Data Analysis

Spectra were analyzed with DIA-NN 1.8.1 [25] using library free search against the UniProt human database (UP000005640, September 2020). Mass accuracy was set to 20 ppm (MS1) and 15 ppm (MS2), respectively, and the maximum number of missed cleavages was set to 1. Precursor FDR was set to 1%. DIA runs from all samples were used to generate a spectral library that was re-applied to all samples using the match-between-runs (MBR) mode. Proteins detected in less than 70% of samples within at least one study group were excluded from further statistical analysis to ensure reliable quantification and comparability between groups.

### 2.3. Functional Annotation and Enrichment Analyses of Differentially Expressed Proteins

Functional annotation and enrichment analyses of dysregulated proteins including Gene Ontology (GO) and Kyoto Encyclopedia of Genes and Genomes (KEGG) pathways were performed using STRING (https://string-db.org, 10 March 2026) [26] and Metascape (http://metascape.org, 10 March 2026) [27]. The proteins identified in EV-enriched fraction were assessed using the ExoCarta database (http://www.exocarta.org, 10 March 2026) [28] to determine their prior annotation as extracellular vesicle-associated proteins.

### 2.4. Statistical Analysis

Significant proteome changes between all four groups were identified via ANOVA with Tukey post-hoc test and among each two groups via unpaired two-sided Student’s *t*-test using Perseus (V.1.6.15.0). Normalized protein abundance values obtained from DIA-NN were log_2_-transformed prior to statistical analysis. Multiple testing correction was applied using permutated-based false discovery rate (FDR) adjustment, and adjusted *p*-values (*q*-values) were considered for the identification of statistically significant protein changes. The FDR and number of randomizations was set to 0.05 and 250, respectively, and S0 to 0.1. Multiple linear regression analysis was performed to assess the potential confounding effects of age, ISS, ASA score, and sex. These analyses were performed only for six preselected candidate proteins identified in the discovery analysis, including two EV-associated proteins and four plasma proteins. All clinical data included in this study were retrospectively extracted from electronic patient records. No additional prospective clinical data collection was performed. Visualization and statistical analysis were performed with GraphPad Prism software (version 10.4.2, GraphPad Software Inc., La Jolla, CA, USA). Data visualization by means of heat map was performed using MetaboAnalyst 6.0 platform [29]. Statistical significance was defined as *p*-value < 0.05.

## 3. Results

### 3.1. Patients

In TBI group the average ISS score was significantly lower and mean age was significantly higher as compared to PT and PT-TBI groups (Table 1) reflecting that this cohort predominantly consisted of older patients with isolated traumatic brain injury.

**Figure 1:**
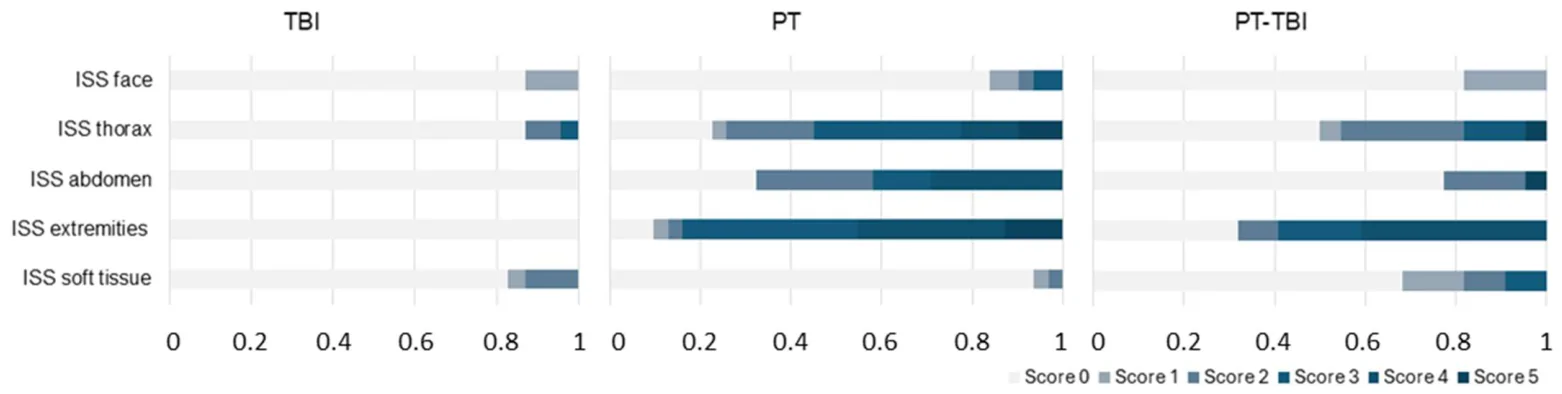
**Overview of injury patterns (scores) across study groups.** The proportion of patients with scores 0–5 in the Injury Severity Score (ISS) for the face, thorax, abdomen, extremities, and soft tissues in each study group. TBI: isolated traumatic brain injury; PT: polytrauma without TBI; PT-TBI: polytrauma with TBI.

This is also reflected in Figure 1, showing injury patterns in study groups. As expected, the lowest mean Glasgow Coma Scale (GCS) score was observed in the PT-TBI group, followed by the TBI group, with both groups significantly different from each other and significantly lower than in the PT group. The mortality rate was also markedly higher in both TBI groups (Table 1).

### 3.2. Overall Trauma Response in Both Compartments

**Figure 2:**
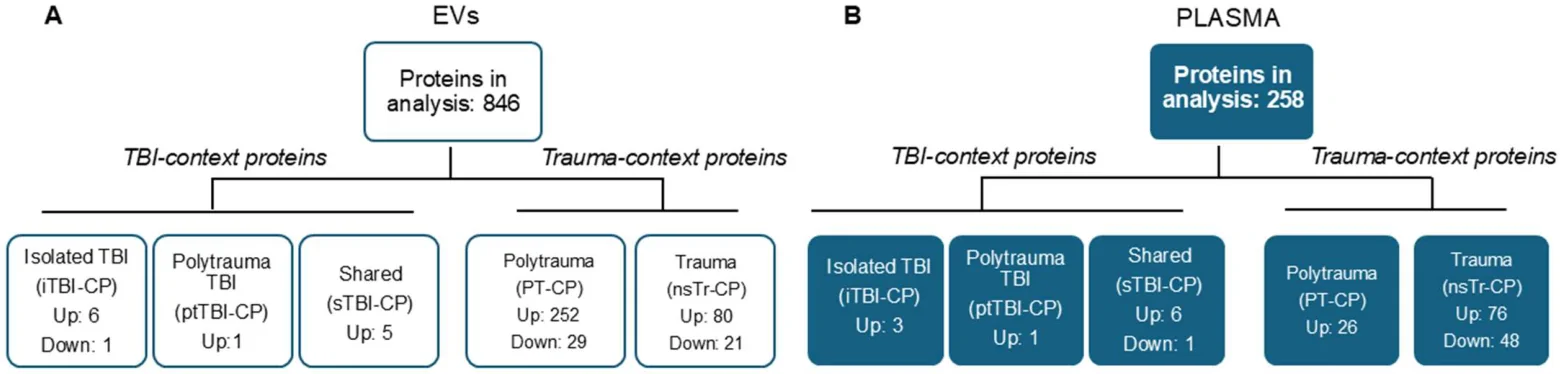
**Overview of proteomic profiles in the EV-enriched plasma fraction (A) and plasma (B).** A total of 846 proteins in the EV-enriched fraction and 258 proteins in plasma were identified and classified according to their differential expression patterns. TBI-context proteins (TBI-CP) comprise proteins associated with isolated TBI (iTBI-CP), polytrauma with TBI (ptTBI-CP), or shared between both TBI groups (sTBI-CP). Trauma-context proteins (Tr-CP) include polytrauma-specific proteins (PT-CP) and non-specific trauma-associated proteins (nsTr-CP). Up and Down indicate the numbers of upregulated and downregulated proteins, respectively.

Comparison of the plasma and EV-enriched plasma fraction proteomes revealed a markedly higher number of proteins with dysregulated expression in one or more patient groups in the EV-enriched fraction than in whole plasma (846 vs. 258; Figure 2), highlighting the increased analytical sensitivity of EV-based proteomic profiling. For further analysis, the identified proteins were categorized into two major groups based on their expression patterns across patient and control cohorts: TBI-context proteins (TBI-CP) and trauma- context proteins (Tr-CP) (Figure 2). Proteins in the TBI-CP group showed significant dysregulation in TBI- and/or PT-TBI patients compared to both healthy controls and polytrauma (without TBI) patients. The Tr-CP category included proteins whose abundance differed significantly from healthy controls but did not differ consistently among the patient groups. Proteins in the TBI-CP were farther sub-divided into three subgroups: (1) isolated TBI-specific context proteins (iTBI-CP, increased/decreased abundance only in isolated TBI patients); (2) PT-TBI-specific context proteins (ptTBI-CP, increased/decreased abundance only in PT-TBI patients) and (3) shared TBI-context proteins (sTBI-CP, increased/decreased abundance in both isolated TBI and PT-TBI patients). Within the Tr-CP category, specific subgroups of non-specific trauma-context proteins (nsTr-CP, differentially expressed across trauma patient groups without group specificity) and polytrauma-context proteins (PT-CP, differentially expressed in polytrauma patients) were identified (Figure 2, Supplementary Figure S1).

Across both proteome datasets, most dysregulated proteins were classified as trauma-context proteins (Tr-CP), whereas only a small subset was assigned to the TBI-context protein (TBI-CP) group (Figure 2). Although the number of proteins in specific sub-categories was comparable between the EV-enriched plasma fraction and plasma proteomes, the overall number of polytrauma context proteins was markedly higher in the EV-enriched proteome. Notably, only in the EV-enriched plasma fraction proteome profile proteins that were downregulated in polytrauma (*n* = 29) were detectable. Overall, most differentially expressed proteins were upregulated compared to healthy controls, with only a few proteins showing downregulation (Figure 2, Supplementary Figure S1). Several proteins were found dysregulated in both EV-enriched fraction and plasma compartments. For example, LCP1 and ENO1 were upregulated in response to polytrauma in EV-enriched fraction and in plasma. In response to trauma, expression of 14 proteins was upregulated, whereas GPX3 and HBB were downregulated in both compartments. Notably, expressions of MINPP1 and LCAT follow opposite regulation pattern in EV-enriched fraction and plasma (Supplementary Figure S1).

Explorative functional enrichment analysis of trauma associated proteins revealed that in response to polytrauma (PT-CP), significantly enriched biological processes included regulation of protein metabolic processes and proteolysis in EV-enriched compartment, and glycolytic processes and ATP metabolic processes in the plasma compartment (Table 2). In response to overall trauma (nsTr-CP), complement activation and blood coagulation processes were significantly enriched in both compartments. Notably the downregulated processes differed between EV- EV-enriched fraction and plasma: in EV these were regulation of protein metabolic processes and regulation of proteolysis, whereas in plasma-complement activation and the acute-phase response were downregulated (Table 2, Supplementary Figures S2–S4).

### 3.3. TBI-Context Proteins in EV-Enriched Fraction and Plasma

**Figure 3:**
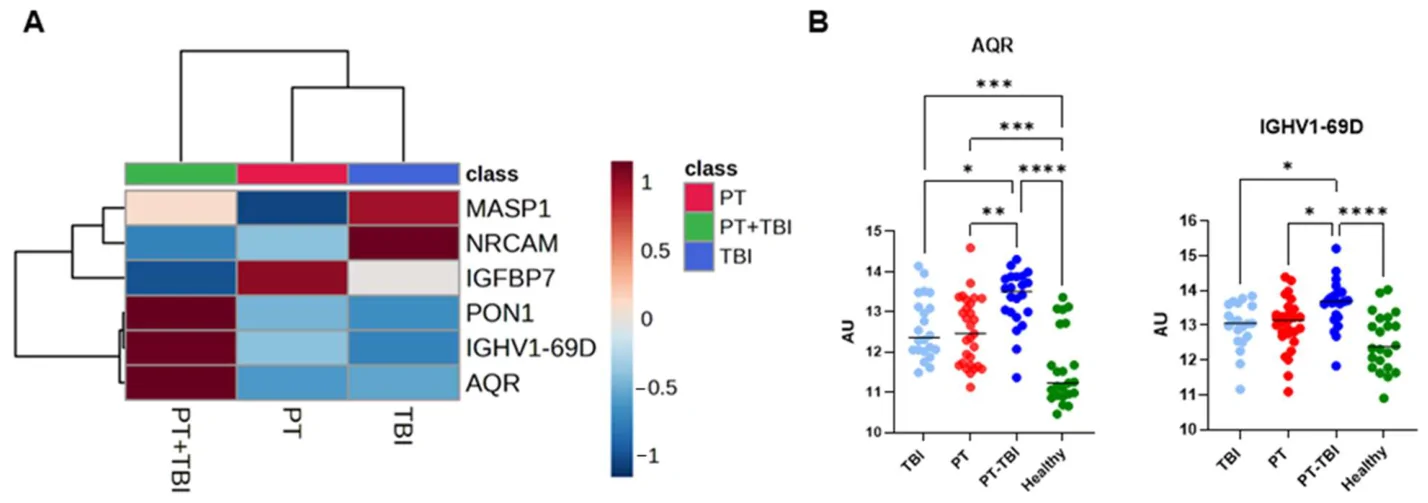
**TBI-context proteins identified in EV-enriched plasma fraction and total plasma.** (**A**) The relative abundance of proteins identified as associated with TBI in the context of polytrauma in the EV-enriched plasma fraction and plasma. Protein abundances are displayed as normalized values, with hierarchical clustering performed on both samples and proteins; group mean profiles are shown alongside the individual samples. (**B**) Differential expression of the candidate proteins AQR (EV-enriched plasma fraction) and IGHV1-69D (plasma). Individual patient values and group means are shown. AU—arbitrary units (protein abundance), * *p* < 0.05; ** *p* < 0.01; *** *p* < 0.001; **** *p* < 0.0001.

Among the TBI-CP proteins in the EV-enriched compartment, most were significantly dysregulated in iTBI-CP group (6 upregulated and 1 downregulated), compared to all other groups and had been previously reported in EVs (according to ExoCarta database), with the exception of NRCAM, NELL2, and VGF, which have not been previously described in EVs (Supplementary Table S1). In the plasma compartment, the largest group consisted of proteins dysregulated in both TBI and PT-TBI patients (sTBI-CP), including 6 upregulated and 1 downregulated protein (Figure 2, Supplementary Table S2). Notably the only one protein in EV-enriched fraction (AQR) and one in plasma (IGHV1-69D) were detected as significantly upregulated in PT-TBI patients (Figure 3). Regarding brain tissue specificity, two brain-specific (NELL2 and NRCAM) proteins in EVs and one CNS-associated protein in plasma (PTGDS) were found to be upregulated in patients with isolated TBI. The protein EFEMP1 with suggested function in glial cells was found to be upregulated in patients with isolated TBI and PT-TBI in EV-enriched compartment and only in patients with isolated TBI in plasma (Table 3 and Supplementary Tables S1 and S2).

In addition, classical TBI markers such as GFAP, NEFL and MAPT were not detected among the dysregulated proteins in either plasma or EV-enriched fraction. Although S100B and ENO2 were detected, they were present in less than 70% of subjects within the same study group and therefore did not meet the predefined filtering criteria.

Regarding the protein functions, among the TBI-context proteins, a notable group was involved in hemostasis and coagulation, with this group being particularly prominent in plasma. The remaining proteins were either associated with basic cellular functions, or their roles have not yet been characterized (Table 3, Supplementary Tables S1 and S2).

Given the significant differences in mean age, ASA score, and ISS among the patient groups, proteins identified as significantly dysregulated in the TBI context were further evaluated for potential confounding effects. As expected, the expression of most proteins was influenced by one or more confounders, or the observed group differences were no longer significant after adjustment for these factors. Only two proteins in the EV fraction (NRCAM and AQR) and four in plasma (IGHV1-69D, MASP1, PON1 and IGFBP7) were not affected by these confounders and were therefore selected for further analysis (Table 3, Supplementary Tables S1 and S2). In Figure 3A, the heat map illustrates the mean expression of these selected proteins across study groups, with hierarchical clustering applied to both protein profiles and groups. Figure 3B shows representative examples of AQR and IGHV1-69D expression levels across the different study groups.

### 3.4. Potential Clinical Relevance of TBI-Context Proteins

To explore the potential clinical relevance of the identified TBI-context proteins, correlation analyses with clinical parameters were performed. All significant correlations with at least a moderate effect size (Spearman coefficient ≥ 0.4) are summarized in Table 4. Among the selected proteins, only those in the EV-enriched fraction were associated with certain clinical parameters, whereas plasma proteins showed no moderate or strong associations with any of the analyzed parameters (Table 4). NRCAM expression showed moderate negative associations with injury-related clinical parameters (ISS and IL-6), as well as with organ-specific biomarkers, including liver-related markers (aspartate aminotransferase [AST] and lactate dehydrogenase [LDH]), a cardiac marker (CK-MB), and a musculoskeletal trauma marker (myoglobin).

### 3.5. Association with Neurological Deterioration and Outcome

**Figure 4:**
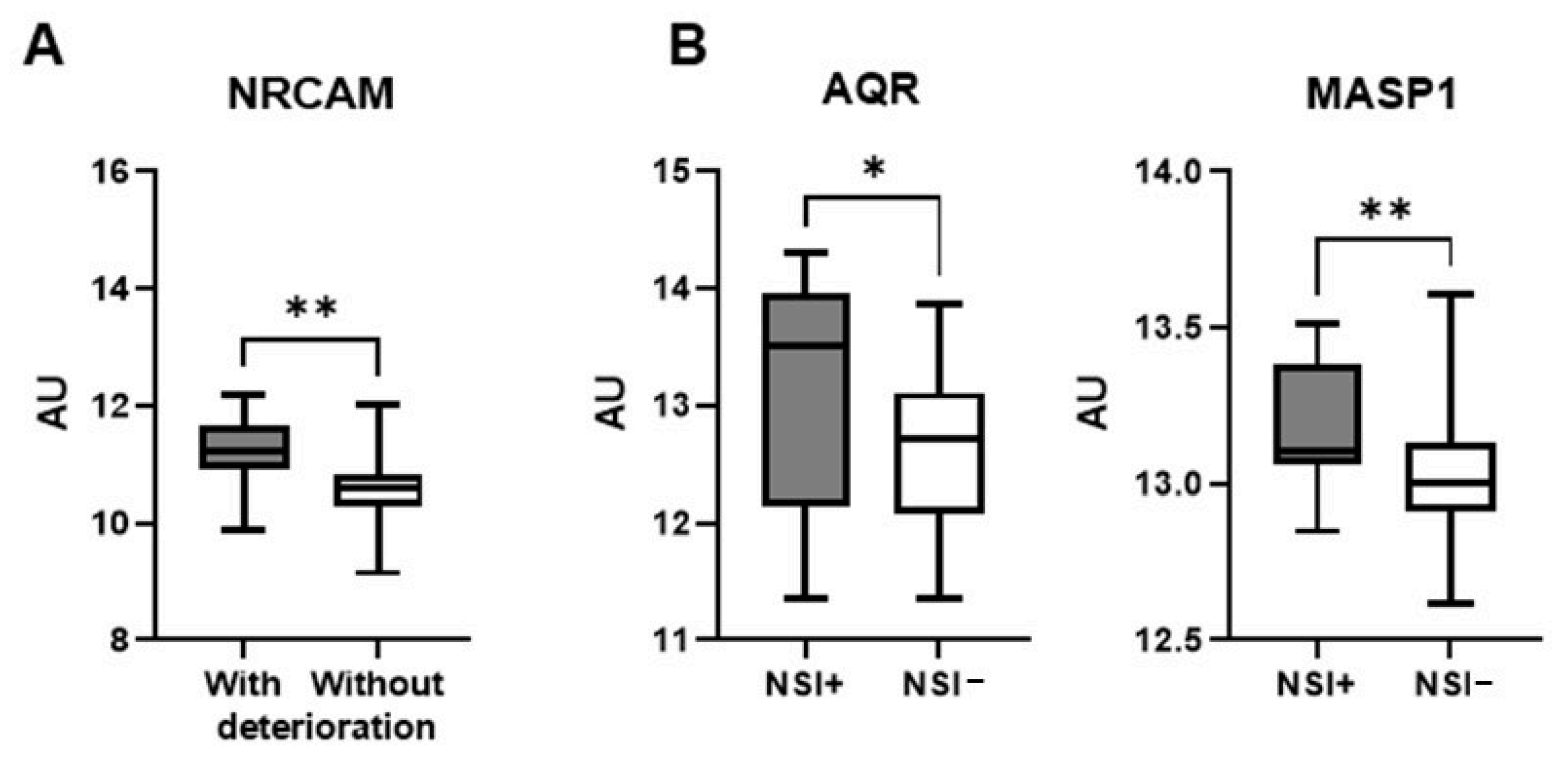
**Association of TBI-context proteins with neurological deterioration.** (**A**) The level of NRCAM protein in EV-enriched plasma fraction was higher in TBI/PT-TBI patients with neurological deterioration, as in TBI/TBI-PT patients without. (**B**) The level of EV-AQR and plasma MASP1 was significantly higher in patients who needed neurosurgery intervention (NSI+).* *p* < 0.05; ** *p* < 0.01.

To analyze if selected TBI-context proteins from both compartments could have prognostic potential, patients with TBI (both groups, isolated TBI and PT-TBI) were stratified according to the neurological status (with *n* = 30/without *n* = 15 neurological deterioration; with *n* = 17/without *n* = 28 neurosurgery intervention) or as survivor (*n* = 37)/non-survivor (*n* = 8). Expression levels of selected proteins were compared in each two groups. Results (Figure 4) showed that EV-associated NRCAM levels were significantly (*p* < 0.01) increased in patients with neurological deterioration.

Patients who needed neurosurgical interventions had significantly higher levels of EV protein AQR (*p* < 0.05) and plasma protein MASP1 (*p* < 0.01). All other proteins showed no significant difference among the stratified subgroups and none of the proteins were associated with (non)survival.

## 4. Discussion

Distinguishing polytrauma with TBI from polytrauma alone is particularly challenging in the emergency setting, where rapid clinical decisions are required. The substantial overlap in biochemical profiles, including systemic cytokine release, endothelial injury, and activation of coagulation pathways limits the usefulness of biomarkers for clear differentiation between polytrauma patients with and without TBI [16]. In severe trauma, blood–brain barrier disruption may occur even without direct brain injury [30], leading to leakage of CNS-associated proteins into the circulation [31]. As a result, so-called “brain injury markers” are not truly brain-specific in the context of severe polytrauma, as systemic inflammation and shock can produce similar signatures [16,32]. In this context, EVs circulating in blood represent a promising alternative source of information, as they carry protected molecular cargo such as proteins, lipids, and nucleic acids that reflect the physiological state of their cells of origin [33]. Importantly, EVs released from neurons and glial cells may provide a more specific and stable signature of central nervous system injury [34], potentially allowing better discrimination between isolated polytrauma and polytrauma with concomitant traumatic brain injury [35]. Therefore, in this study, we compared the proteomic profiles of total plasma and the EV-enriched plasma fraction among patients with isolated TBI, polytrauma with concomitant TBI, and polytrauma without TBI at an early time point (emergency room admission). These profiles were further compared with those of healthy controls.

As hypothesized, our results showed that the EV compartment appears to respond more strongly to trauma overall, as a greater number of proteins were dysregulated compared with plasma. This increased degree of proteomic alteration also translated into compartment-specific functional patterns. Whereas the overall response to trauma appears to involve overlapping pathways in both compartments, such as complement activation and coagulation, the protein profile changes associated with polytrauma differed between plasma and the EV-enriched fraction. Specifically, proteome changes in plasma following polytrauma were associated with energy metabolism, including ATP metabolic processes and glycolysis, whereas in the EV compartment, altered proteins were predominantly linked to protein metabolic processes and proteolysis. These differences may reflect distinct roles of the two compartments in the systemic response to polytrauma, with plasma likely capturing a rapid, systemic metabolic adaptation to injury, while EVs may reflect cellular responses and intercellular communication. However, as the analyzed material represents an EV-enriched fraction rather than a purified EV population, the contribution of co-isolated plasma proteins to the observed proteomic profiles cannot be excluded.

Regarding proteins dysregulated in context of TBI the number of identified proteins was similarly low in both compartments in the systemic circulation, further underscoring the well-known challenges of biomarker-based TBI diagnosis in the setting of polytrauma. Among the TBI-context proteins, brain-specific proteins (NRCAM and NELL2) were detected exclusively in the EV-enriched fraction. This observation together with our previous findings [17] further supports that the EV compartment may more specifically reflect TBI-related processes and therefore could provide a more sensitive source of TBI- biomarkers. As this pilot study included relatively small patient cohorts with notable differences in clinical and demographic characteristics between groups, the expression of some TBI-context proteins was found to be confounded either by ISS, age or ASA. These proteins were therefore excluded from further characterization in the present study; however, they remain of interest and warrant investigation in future studies with larger, better-matched patient cohorts.

Expression of two proteins in EV compartment (NRCAM, AQR) and four (IGHV1-69D, MASP1, PON1 and IGFBP7) in plasma was not confounded by ISS, age or ASA. NRCAM is a neuronal cell adhesion molecule of the L1 immunoglobulin superfamily involved in neural development, axon growth and guidance, synapse formation, and myelination [36]. NRCAM was recently identified in human brain tissue–derived EVs [37]. Increased levels of NRCAM+ EVs have also been reported in brain tissue and plasma in a chronic restraint stress mouse model [38]. Although neuron-derived EVs have been studied in TBI, NRCAM has not previously been used as a marker. Our findings show increased NRCAM levels in the EV-enriched plasma fraction in TBI patients compared with polytrauma patients and healthy controls. Levels were further elevated in TBI patients with neurological deterioration, suggesting that NRCAM^+^ EVs may reflect neuronal injury and represent a potential TBI biomarker.

AQR (Aquarius intron-binding spliceosomal factor) is an RNA splicing factor involved in RNA processing, genome stability, and DNA damage [39,40]. It has been consistently detected in EVs from multiple sources, including plasma, brain tissue, and cancer cells, supporting its potential as a circulating biomarker. AQR has also been linked to nerve injury responses, metabolic regulation, vascular integrity, aging, and neurodegenerative diseases, including Alzheimer’s disease [39,41,42]. Together with our findings showing correlations between EV-AQR levels, GCS, and the need for neurosurgical intervention, these data suggest that EV-AQR may represent a multifunctional biomarker candidate in TBI.

IGHV1-69D (immunoglobulin heavy variable 1–69D), is part of the antibody variable region repertoire, often reflecting B-cell activation and humoral immune responses [43,44]. It was found to be significantly enriched in plasma of patients with early neurological improvement after stroke (transcriptomic study) [45]. Our findings of selective IGHV1-69D upregulation in PT-TBI may indicate a synergistic immune response, where simultaneous central nervous system injury and peripheral trauma amplify adaptive immune activation beyond levels seen in isolated TBI or polytrauma. Future studies should evaluate the temporal dynamics of IGHV1-69D expression after injury to determine whether its upregulation persists during later phases of recovery and whether it is associated with neurological outcome, systemic inflammation, or secondary injury processes.

MASP-1 mannose-binding lectin (MBL)-associated serine protease-1 activates the lectin pathway of the complement system and mediates both proinflammatory and procoagulant responses. It has been proposed as a biomarker of disease severity in trauma and sepsis [46], while lectin pathway activation is implicated in post-traumatic neuroinflammation and secondary injury after TBI [47,48]. Our finding of elevated plasma MASP-1 levels in isolated and combined polytrauma TBI, particularly in patients requiring neurosurgical intervention, supports its association with TBI severity and outcome and further highlights its role in the interplay between complement activation, inflammation, and coagulation after TBI.

Paraoxonase 1 (PON1) is an antioxidant enzyme primarily synthesized in the liver that protects against vascular, neurological, and kidney injury by reducing oxidative stress and inflammation [49]. Recently it was suggested as a novel therapeutic agent against ischemia-reperfusion injury in cerebral infarction [50]. Polymorphism in PON1 gene was associated with encephalopathy development after TBI [51]. The increased levels of PON1 in TBI and PT-TBI compared to polytrauma and healthy controls may reflect an enhanced systemic antioxidant response to brain injury–associated oxidative stress. As an HDL-associated enzyme with anti-oxidative and anti-inflammatory properties, PON1 upregulation may represent a compensatory mechanism aimed at limiting lipid peroxidation and secondary tissue damage following neurotrauma.

IGFBP7 Insulin-like growth factor-binding protein 7, is highly enriched in activated vasculature during development, physiological and pathological tissue remodeling. Despite all efforts invested into researching IGFBP7, the biological function of this protein is not well understood and is seen as microenvironment dependent [52]. Recent transcriptomic study found IGFBP7 to be upregulated in vasculature in response to TBI in mouse model and surgical samples from patients with brain injury [53]. The increased expression of IGFBP7 in TBI and PT-TBI, but not in isolated polytrauma, suggests a neurotrauma-specific activation of endothelial stress and blood–brain barrier dysfunction pathways. As a regulator of vascular integrity and cellular stress responses, IGFBP7 may reflect neurovascular injury and systemic stress amplification associated with traumatic brain injury.

Correlation analysis of selected proteins from EV-enriched plasma fraction and plasma with clinical parameters revealed that none of the TBI-context proteins identified in the plasma compartment were significantly associated with any of the measured variables, whereas EV-fraction proteins were associated with several parameters. The lack of correlation in plasma, contrasted with significant associations observed in EVs, suggests that EV-enriched fractions may provide complementary information regarding injury severity and organ involvement. However, this finding should be interpreted cautiously, as limited statistical power may contribute to the observed differences, and further studies are required to confirm whether proteins from EV-enriched plasma fraction provide distinct biological information compared with total plasma proteins.

Notably, classical TBI injury markers such as S100B, GFAP, and ENO2 were either not detected among the dysregulated proteins in plasma or the EV-enriched fraction or were excluded from further analysis due to failure to meet the predefined filtering criteria. This likely reflects the limited sensitivity of untargeted proteomics for low-abundance (CNS-derived) proteins in the context of severe systemic injury, where inflammatory and multi-organ signals may predominate. Nevertheless, the EV-enriched fraction showed a broader representation of neuro-associated proteins (e.g., NELL2, NRGN, NRCAM, NCAN, VGF and NRP2) compared with plasma, which contained only a limited subset (e.g., NRP1). While these findings do not indicate selective enrichment of established CNS injury markers, they suggest that EV-enriched plasma profiling may provide additional information on neuro-related molecular changes in the setting of polytrauma.

This study has several limitations that should be considered when interpreting the findings. The comparison of isolated TBI and polytrauma groups reflects the clinical complexity of these distinct patient populations, including expected differences in age, ISS, and AIS. Although statistical approaches were applied to account for relevant confounding factors, future studies with larger cohorts will be necessary to further investigate these effects. In addition, samples were analyzed at a single early time point after injury, providing insights into the acute phase but not capturing the longitudinal dynamics of proteomic alterations. Future longitudinal studies will help to further characterize temporal changes in plasma and EV-associated protein profiles. Furthermore, the used strategy generates an EV-enriched fraction, which enables investigation of vesicle-associated molecular signatures but may also contain co-isolated non-vesicular proteins. Finally, as this study represents a discovery-oriented proteomic analysis, the identified candidate biomarkers require validation in independent cohorts. Together, these findings provide a foundation for future studies aimed at validating and further developing EV-associated biomarkers in traumatic injury.

In summary, this pilot proteomic study demonstrates distinct molecular profiles in the EV-enriched plasma fraction compared with total plasma and identifies candidate TBI-associated proteins in polytrauma. These findings support further validation of EV-based biomarkers in larger, well-characterized cohorts.

## 5. Conclusions

This pilot study demonstrates that EV-enriched plasma proteomics increases the detection of trauma-related proteomic changes compared with whole plasma and identifies biological pathways not detected by conventional plasma analyses. Although most alterations reflected systemic responses to polytrauma, a subset of EV- and plasma-associated proteins showed specific associations with TBI independent of major clinical confounders. In particular, EV-associated NRCAM and AQR, together with plasma MASP1, were associated with neurological status and injury severity in this cohort. These findings identify these proteins as candidate biomarkers for TBI in the setting of polytrauma and warrant further validation in larger, independent patient cohorts.

## Figures and Tables

**Table 1:** Patients’ groups-demographic and clinical data.

Parameter	TBI (*n* = 23)	PT (*n* = 30)	PT-TBI (*n* = 22)	Healthy (*n* = 24)
Male sex	17 (78%)	20 (67%)	19 (86%)	11 (46%)
Age (years)	62 ± 13	45 ± 15 ***	45 ± 14 ***	39 ± 12 ****
ASA	2 (1–3)	1 (1–2)	1 (1–2)	N/A
Injury Severity Score (ISS)	20 ± 6	29 ± 10 **	31 ± 10 ***	N/A
Glasgow Coma Scale (GCS)	11 ± 5	14 ± 2 *	7 ± 5 *^(####)^	N/A
Mortality	4 (17%)	1 (3.3%)	4 (18%)	N/A

* *p* < 0.05; ** *p* < 0.01; *** *p* < 0.001; **** *p* < 0.0001 versus TBI; ^(####)^ *p* < 0.0001 PT vs. PT-TBI, N/A—non applicable.

**Table 2:** Functional enrichment of dysregulated proteins in trauma-derived EVs and plasma (STRING web interface).

Proteome		Regulation	Biological Processes (Gene Ontology, Top 5)
EV	nsTr-CP	up	GO:0006956 Complement activation GO:0019835 Cytolysis GO:0007596 Blood coagulation
		down	No enriched processes
	PT-CP	up	GO:0051246 Regulation of protein metabolic processes GO:0046907 Intracellular transport GO:0030162 Regulation of proteolysis GO:0051649 Establishment of localization in cell GO:0007010 Cytoskeleton organization
		down	GO:0030527 Structural constituents of chromatin GO:0005198 Structural molecule activity
Plasma	nsTr-CP	up	GO:006956 Complement activation GO:0007596 Blood coagulation GO:0072378 Fibrin clot formation GO:0042730 Fibrinolysis
		down	GO:0006956 Complement activation GO:0006953 Acute-phase response GO:0043691 Reverse cholesterol transport GO:0042744 Hydrogen peroxide catabolic process GO:0002252 Immune effector process
	PT-CP	up	GO:0006096 Glycolytic process GO:0046034 ATP-metabolic process GO:005996 Monosaccharide metabolic process GO:0071803 Positive regulation of podosome assembly GO:0043254 Regulation of protein-containing complex assembly
		down	No proteins

nsTr-CP—non-specific trauma context proteins; PT-CP—polytrauma context proteins.

**Table 3:** EV and plasma proteins associated with TBI: functional annotations.

	ID (Name)	Tissue/Cell Type Enriched	Functions
EV	Upregulated, iTBI-CP		
	NRCAM (Neuronal cell adhesion molecule)	Brain Oligodendrocyte precursor cells, excitatory neurons, astrocytes, inhibitory neurons	Neurite outgrowth; Schwann cell–axon interactions; cell adhesion
	Upregulated, ptTBI-CP		
	AQR (RNA helicase Aquarius)	Non-specific	Component of the spliceosome
PLASMA	Upregulated, ptTBI-CP		
	IGHV1-69D (Immunoglobulin heavy variable 1-69D)	Plasma cells, lymphoid tissue	V region of the variable domain of immunoglobulin heavy chains that participates in the antigen recognition
	Upregulated, sTBI-CP		
	MASP1 (Mannan-binding lectin serine protease 1)	Tissue enhanced: cervix, heart muscle, liver	Precursor of a serum protease involved in activation of the complement system, promoting pathogen clearance and immune signaling
	PON1	Tissue enriched: liver	Hydrolyzes toxic metabolites of organophosphorus insecticides; protects LDL from oxidative modification and atheroma formation.
	(Serum paraoxonase/arylesterase 1)		
	Downregulated, sTBI-CP		
	IGFBP7 (Insulin-like growth factor-binding protein 7)	Tissue enhanced: blood vessel	Binds IGF1 and IGF2 with low affinity; stimulates prostacyclin (PGI2) production and cell adhesion; ligand for CD93 involved in angiogenesis

**Table 4:** Correlation analysis of EV und plasma proteins expression levels and clinical data.

Compartment/Protein		Parameter	Spearman	*p*-Value
EV	Upregulated, iTBI-CP			
		ISS (Injury Severity Score)	−0.48	<0.0001
	NRCAM	IL-6 (Interleukin 6)	−0.42	0.0002
		AST (Aspartate Aminotransferase)	−0.46	0.0006
		LDH (Lactate Dehydrogenase)	−0.49	0.0003
		CKMB (Creatine Kinase–MB isoenzyme)	−0.45	0.0009
		Myoglobin		
			−0.50	0.0002
	Upregulated, ptTBI-CP			
	AQR	GCS (Glasgow Coma Scale)	−0.51	<0.0001
		Troponin T	0.50	0.036
		Myoglobin	0.45	0.0012
Plasma	Upregulated, ptTBI-CP			
	IGHV1-69D	-	-	-
	Upregulated, sTBI-CP			
	MASP1	-	-	-
	PON1	-	-	-
	Downregulated, sTBI-CP			
	IGFBP7	-	-	-

Notably, upregulated in PT-TBI patients’ EV-enriched fraction AQR protein showed moderate negative correlation with GCS and positive moderate correlations with heart marker troponin T and myoglobin.

## Data Availability

Raw data files of all samples processed in the proteomics analysis are hosted on the PRoteomics IDEntifications Database (PRIDE) under the following URL: http://www.ebi.ac.uk/pride (accessed on 24 June 2026), project PXD079127. (Reviewer access: PXD079127 (login name) Token: z6de3VGbQDsq (password)).

## Supplementary Materials

The following supporting information can be downloaded at: https://www.mdpi.com/article/10.3390/medsci14040429/s1, Figure S1: Number of proteins dysregulated in response to overall trauma and polytrauma in EV and plasma compartments; Figure S2: Functional enrichment analysis of upregulated trauma context proteins performed using Metascape; Figure S3: Functional enrichment analysis of upregulated polytrauma context proteins performed using Metascape; Figure S4: Functional enrichment analysis of downregulated trauma context proteins performed using Metascape; Table S1: TBI-context proteins detected in EV compartment, functional annotations; Table S2: TBI-context proteins from plasma compartment, functional annotations.

## Abbreviations

The following abbreviations are used in this manuscript:

TBI	Traumatic brain injury
EV	Extracellular vesicle
ISS	Injury Severity Score
AIS	Abbreviated Injury Scale
PT	Polytrauma
HPLC-MS/MS	High-performance liquid chromatography–tandem mass spectrometry
DEP	Differentially expressed protein
TR-DGU^®^	Traumaregister DGU^®^
SCI	Spinal cord injury
ER	Emergency room
ACN	Acetonitrile
GO	Gene ontology
KEGG	Kyoto Encyclopedia of Genes and Genomes
GCS	Glasgow coma scale
TBI-CP	TBI-context proteins
Tr-CP	Trauma- context proteins
iTBI-CP	Isolated TBI-specific context proteins
ptTBI-CP	PT-TBI-specific context proteins
sTBI-CP	Shared TBI-context proteins
nsTr-CP	Non-specific trauma-context proteins
PT-CP	Polytrauma-context proteins
AST	Aspartate aminotransferase
LDH	Lactate dehydrogenase
CK-MB	Creatine Kinase–MB isoenzyme
PRIDE	Proteomics identifications Database
