# Supplementary material for: Traumatic Brain Injury in the Omics Era: Plasma and Extracellular Vesicle Proteomic Signatures in Polytrauma
Source: Med Sci (Basel). 2026 Jul 25;14(4):429. doi: 10.3390/medsci14040429 (PMC13515644; doi:10.3390/medsci14040429)
Supplement: Supplementary file 1 [file medsci-14-00429-s001.zip › medsci-4427168-supplementary.pdf]

Supplementary Data

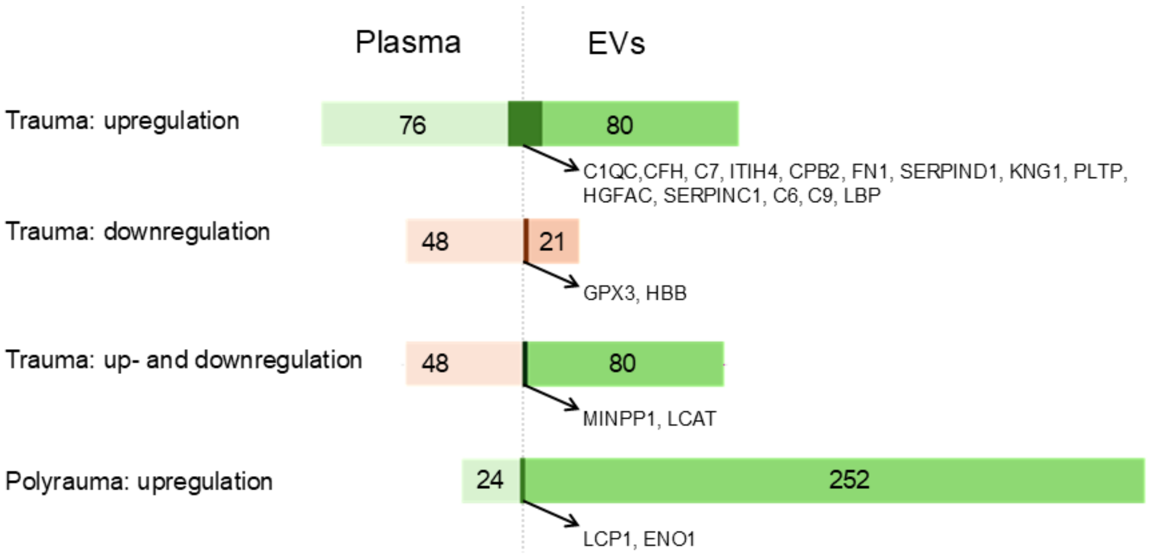

Figure S1. Number of proteins dysregulated in response to overall trauma and polytrauma in EV and plasma compartments**.** The trauma-context category includes proteins significantly dysregulated in patients compared to healthy controls but without consistent differences between patient subgroups. The polytrauma-context subset comprises proteins significantly dysregulated in both PT and PT-TBI patients compared with controls and TBI patients. Green indicates upregulated proteins and rose indicates downregulated proteins. Numbers inside the bars and the length of the bars represent the number of dysregulated proteins in each compartment. Proteins dysregulated in both compartments are listed below the corresponding bars and indicated by arrows.

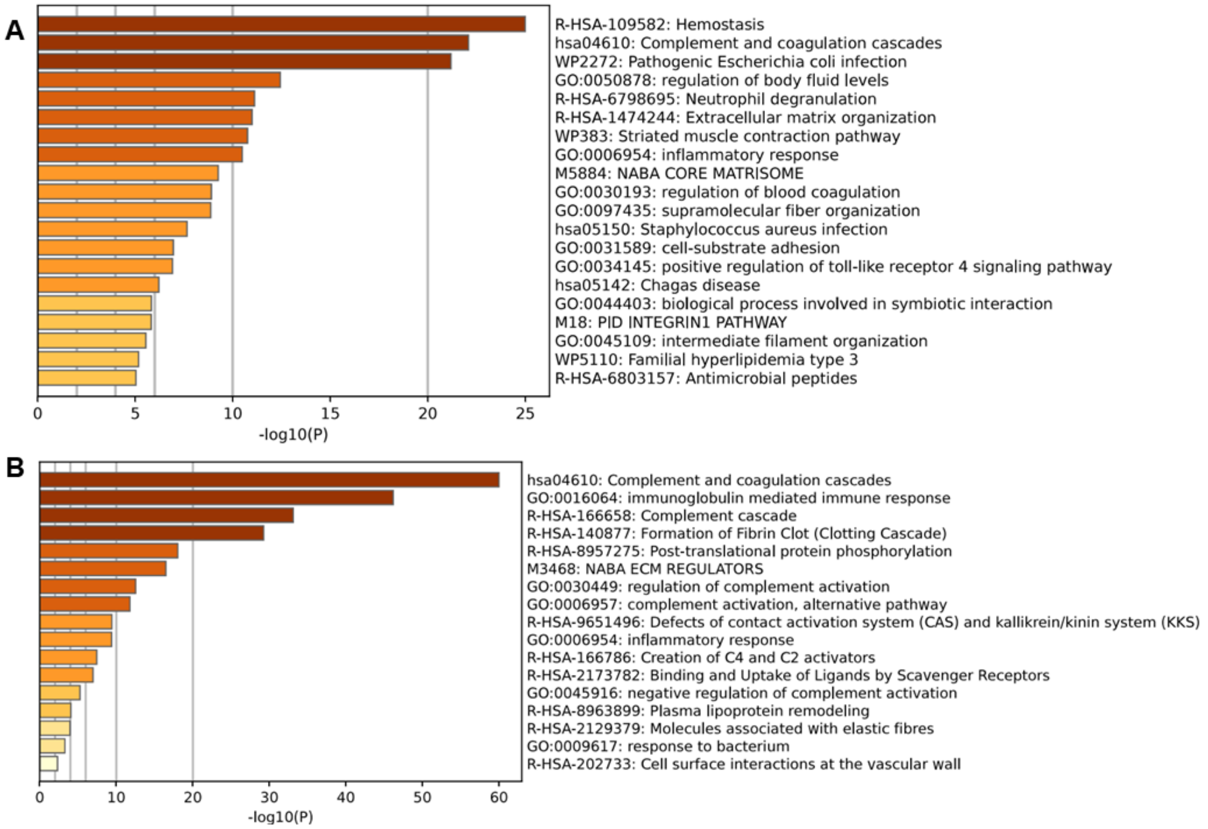

Figure S2. Functional enrichment analysis of upregulated trauma context proteins performed using Metascape. The top 20 significantly enriched pathways for EV (A) and plasma (B) proteins are shown. Bar length represents enrichment significance expressed as −log10(P value). Pathways are ranked according to statistical significance**.**

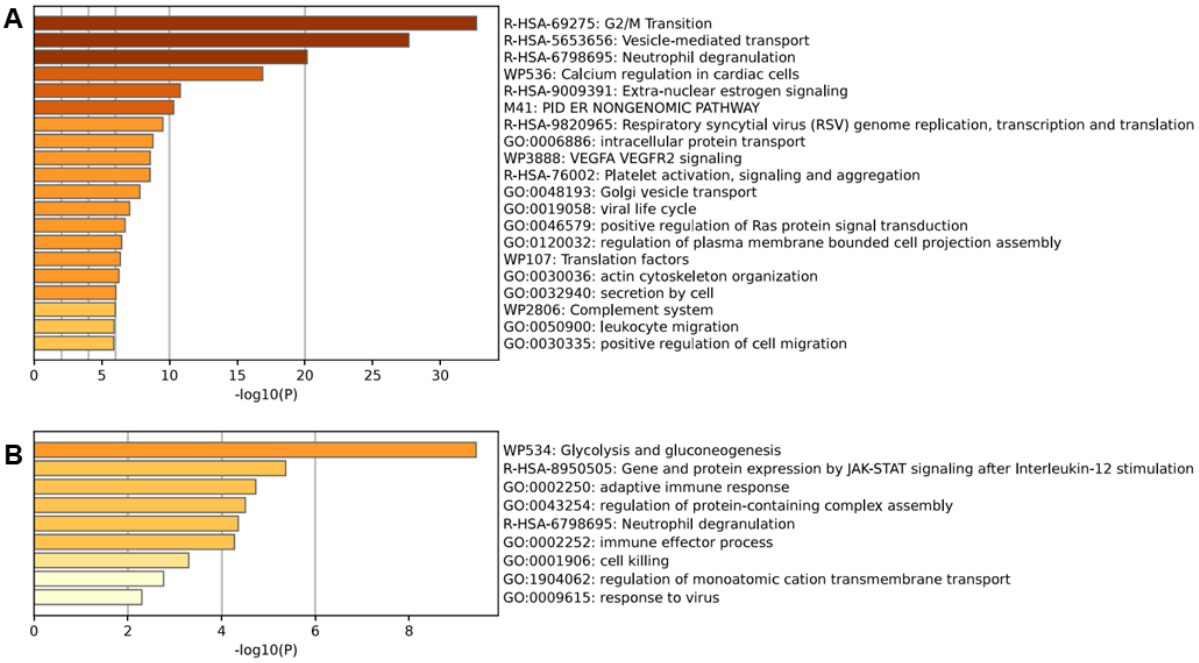

Figure S3. Functional enrichment analysis of upregulated polytrauma context proteins performed using Metascape. The top 20 significantly enriched pathways for EV (A) and plasma (B) proteins are shown. Bar length represents enrichment significance expressed as −log10(P value). Pathways are ranked according to statistical significance**.**

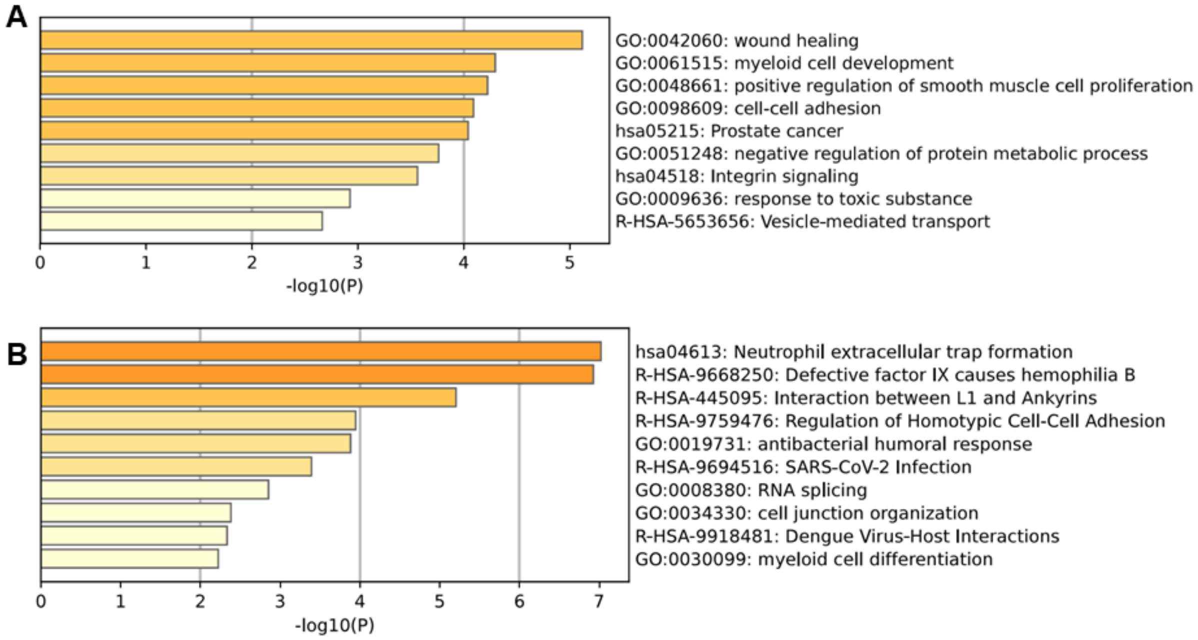

Figure S4. Functional enrichment analysis of downregulated trauma context proteins performed using Metascape. The top 20 significantly enriched pathways for EV (A) and plasma (B) proteins are shown. Bar length represents enrichment significance expressed as −log10(P value). Pathways are ranked according to statistical significance.

Table S1. TBI-context proteins detected in EV compartment, functional annotations.

ID/ Name	ExoCarta	Tissue/Cell type enriched	Functions (UniProt)	Multivariable linear regression (Group/Confounder)
iTBI-CP, upregulation				
NRCAM (Neuronal cell adhesion molecule)	-	Brain, Oligodendrocyte precursor cells, excitatory neurons, astrocytes, inhibitory neurons	Neurite outgrowth; Schwann cell–axon interactions; cell adhesion	***/-
NELL2 (Protein kinase C-binding protein NELL2)	-	Brain, Choroid plexus, retina	Required for neuronal survival via modulation of MAPK signaling pathways (by similarity)	ns/ISS
FGA (Fibrinogen α chain)	+	Liver, plasma and extracellular compartments	Key roles in hemostasis and antimicrobial defense; forms fibrin with FGB and FGG; stabilizes clots	ns/Age, ISS
FGB (Fibrinogen β chain)	+			ns/-
FGG	+			ns/-

(Fibrinogen γ chain)			and supports wound repair and cell migration.	
SVEP1<br>(Sushi, von Willebrand factor type A, EGF and pentraxin domain-containing protein 1)	+	Oligodendrocytes, fibroblasts, mesothelial cells, syncytiotrophoblasts, inhibitory neurons, Leydig cells	Promotes platelet activation	ns/AGE, ISS
iTBI-CP, downregulation				
SND1<br>(Staphylococcal nuclease domain-containing protein 1)	+	Low tissue specificity	Endonuclease mediating miRNA decay of both protein-free and AGO2-bound miRNAs	ns/-
ptTBI-CP, upregulation				
AQR<br>(RNA helicase Aquarius)	+	Non-specific	Component of the spliceosome	**/-
sTBI-CP, upregulation				
EFEMP1<br>(EGF containing fibulin extracellular matrix protein 1)	+	Extracellular positivity in most tissues	May regulate glial cell migration and differentiation and support neuronal neurite outgrowth	*/ISS
BMP1<br>(Bone morphogenetic protein 1)	+	Cytoplasmic expression in most tissues	Plays a key role in regulating extracellular matrix formation	*/ISS
CPN2<br>(Carboxypeptidase N subunit 2)	+	Liver/Located in blood microparticle and extracellular exosome	Predicted to be involved in regulation of catalytic activity	ns/Gender, ISS
FBLN5<br>(Fibulin-5)	+	Extracellular matrix and tissue stroma	May play a role in vascular development and remodeling	***/ISS
VGF<br>(Neurosecretory protein VGF)	-	Neuroendocrine cells subpopulation	Unknown	ns/-

*p<0.05; **p<0.01; ***p<0.001. ns-nonsignificant. ISS-injury severity score.

Table S2. TBI-context proteins from plasma compartment, functional annotations.

ID (Name)	Tissue/Cell type enriched	Functions	Multivariable linear regression (Group/Confounder)
iTBI-CP, upregulation			

PTGDS (Prostaglandin-H2 D-isomerase)	Most tissues; enhanced in: Brain, heart muscle, Testis	Involved in CNS functions (sedation, NREM sleep, PGE2-induced allodynia); may have anti-apoptotic effects in oligodendrocytes and roles in CNS maturation and maintenance	**/AGE
EFEMP1 (EGF-containing fibulin-like extracellular matrix protein 1)	Enhanced in blood vessels	May regulate cell adhesion and migration; in the olfactory epithelium, may also modulate glial cell migration, differentiation, and support of neuronal neurite outgrowth	ns/AGE, ISS
HRG (Histidine-rich glycoprotein)	Plasma; Liver	Plasma glycoprotein binding multiple ligands (heme, heparin, heparan sulfate, thrombospondin, plasminogen, metal ions); involved in immune complex and pathogen clearance, chemotaxis, cell adhesion, angiogenesis, coagulation, and fibrinolysis	ns/ISS
ptTBI-CP, upregulation			
IGHV1-69D (Immunoglobulin heavy variable 1-69D)	Plasma cells, Lymphoid tissue	V region of the variable domain of immunoglobulin heavy chains that participates in the antigen recognition	*/ns
sTBI-CP, upregulation			
F5 (Coagulation factor V)	Enriched in choroid plexus, liver, placenta	Essential cofactor for factor Xa prothrombinase activity, enabling activation of prothrombin to thrombin	ns/Age, ISS
MCAM (Melanoma adhesion molecule)	Endothelial cells, adipocytes and cerebellum	Plays a role in cell adhesion and maintenance of endothelial cell–cell junctions in vascular tissue	ns/Age, ISS
FCGBP (IgGFc-binding protein)	Tissue enhanced: Intestine, Salivary gland, Thyroid gland	May contribute to maintenance of mucosal structure as a gel-like component of mucosal surfaces.	ns/-
MASP1 (Mannan-binding lectin serine protease 1)	Tissue enhanced: cervix, heart muscle, liver	Precursor of a serum protease involved in activation of the complement system, promoting pathogen clearance and immune signaling	*/-
HABP2 (Factor VII-activating protease/Hyaluronan binding protein 2)	Tissue enriched: liver	Does not cleave prothrombin or plasminogen; converts pro-urokinase to active two-chain urokinase	*/ISS

PON1<br>(Factor VII-activating protease/Hyaluronan binding protein 2)	Tissue enriched: liver	Hydrolyzes toxic metabolites of organophosphorus insecticides; protects LDL from oxidative modification and atheroma formation.	***/-
sTBI-CP, downregulation			
IGFBP7<br>(Insulin-like growth factor-binding protein 7)	Tissue enhanced: blood vessel	Binds IGF1 and IGF2 with low affinity; stimulates prostacyclin (PGI2) production and cell adhesion; ligand for CD93 involved in angiogenesis	*/-

*p<0.05; **p<0.01; ***p<0.001. ns-nonsignificant. ISS-injury severity score.
